# WHO NEEDS THEIR NEIGHBORS? EXPLORING NEIGHBORHOOD DISPARITIES IN COGNITIVE FUNCTION THROUGH PATH ANALYSIS

**DOI:** 10.1093/geroni/igac059.2070

**Published:** 2022-12-20

**Authors:** Daniel Rong Yao Gan, John Best

**Affiliations:** Simon Fraser University, Vancouver, British Columbia, Canada; Simon Fraser University, Vancouver, British Columbia, Canada

## Abstract

Neighborhoods are diverse and may or may not present opportunities for stress reduction or social engagement depending on their qualities. Based on the stress connectome (Dum et al., 2019), psychosocial stress may accelerate cognitive aging, which explains place-based disparities in cognitive function. This study examines two attributes of the neighbourhood environment, and their potential to influence cognitive function through semantic fluency.Using aggregated baseline (cross-sectional) data from N=1,010 neighborhoods with 5 or more respondents in the Canadian Longitudinal Study on Aging, we examined the effects of neighborhood greenness and cohesion on aggregates of age-, sex-, and education-adjusted cognitive test scores. Participants were community-dwelling adults aged 44 and above. Semantic fluency was assessed using the Animal Fluency Test (AFT). Delayed recall was assessed using Rey’s Auditory Verbal Learning Test (RAVLT), whereas executive function was assessed using Mental Alternation Test (MAT). Neighborhood qualities were found to affect delayed recall (B=.34, p<.001) and executive function (B=.42, p<.001) through semantic fluency (B=.08, .10, p<.01). Semantic fluency fully mediated the effects of neighborhood attributes on cognitive function. Further stratifying these neighborhoods by socioeconomic status showed that cohesion has stronger effects in poorer neighborhoods (B indirect=.104) than richer neighborhoods (B indirect=.066). The effect of greenness was no longer significant upon stratification. Neighborhoods offer an important social arena for adults in mid- and late-life to practice conversing, especially in poorer neighborhoods, which improves cognitive function. Creating opportunities for socialization by improving cohesion and neighborhood parks may reduce place-based disparities in cognitive health. Causality remains to be ascertained.

